# Deep-learning based adaptive fusion of CC and MLO views for improved mammographic cancer diagnosis

**DOI:** 10.1016/j.mex.2026.103827

**Published:** 2026-02-14

**Authors:** Sannasi Chakravarthy Surulimani Ramaraj, Harikumar Rajaguru, Rajesh Kumar Dhanaraj, Anto Lourdu Xavier Raj Arockia Selvarathinam, Dragan Pamucar

**Affiliations:** a Department of Electronics and Communicaiton Engineering, Bannari Amman Institute of Technology, Sathyamangalam 638 401, India; b Symbiosis Institute of Computer Studies and Research (SICSR), Symbiosis International (Deemed University), Pune, India; c Department of Data Science and Analytics, College of Computing, Grand Valley State University, MI, USA; d Sustainability Competence Centre, Széchenyi István University, Győr, Hungary

**Keywords:** Breast cancer, Craniocaudal, Mammogram, Transfer learning, Deep learning, Explainable AI, Attention

## Abstract

Breast cancer remains the most prevalent malignancy among women worldwide. The timely detection of this cancer type is critical for improving survival outcomes. Despite advancements, mammogram classification using deep learning strategies still faces challenges. These include inter-view feature inconsistency, loss of diagnostic details, and limited interpretability. In order to address these issues, MammoFusion-Net, a dual-branch deep learning framework, is proposed for mammogram-based breast cancer classification. Using residual convolutional streams, the framework processes craniocaudal (CC) and mediolateral oblique (MLO) views independently. This supports preservation of view-specific anatomical information. In the proposed framework, a Gates Cross-View Fusion mechanism adaptively integrates features across views. As a result of experimental analysis, the proposed framework achieved 92.116 % (VinDr-Mammo dataset) and 95.556 % (INBreast dataset) of improved classification performance.

• Employs a dual-branch architecture to independently process CC and MLO views using residual convolutional streams.
• Integrates Gated Cross-View Fusion and attention mechanisms adaptively and refines multi-view features for stronger discrimination.
• Demonstrates the explainability of the model through Grad-CAM visualizations that highlight lesion-relevant regions.

## Graphical abstract

**Figure:**
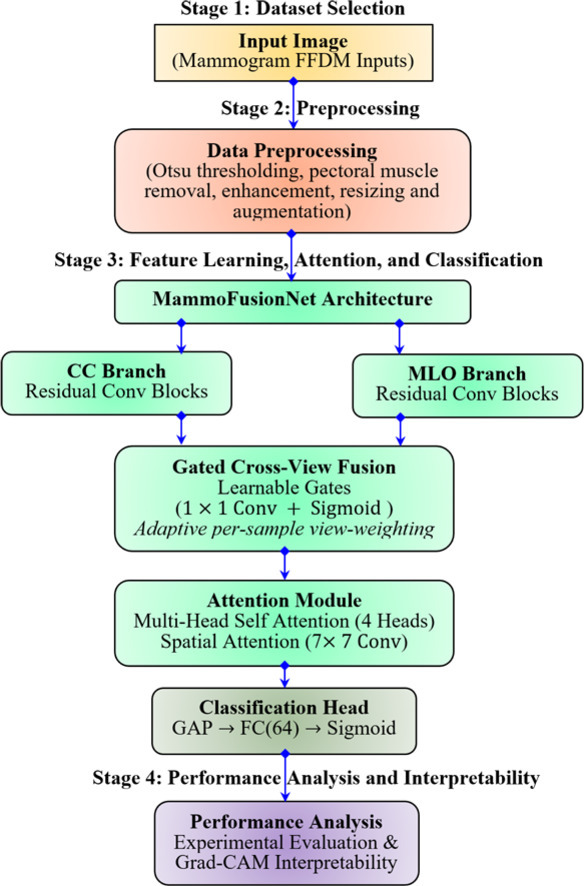

**Specifications table**

**Table:** 

**Subject area**	Engineering
**More specific subject area**	*The paper presents a dual-view mammogram classifier that uses adaptive fusion and attention mechanisms for highly accurate and interpretable breast cancer detection.*
**Name of your method**	*Deep-Learning based Adaptive Fusion of CC and MLO Views for Improved Mammographic Cancer Diagnosis.*
**Name and reference of original method**	*None*
**Resource availability**	*Mammogram Imagery Datasets* The VinDr-Mammo dataset can be publicly accessible at https://www.physionet.org/content/vindr-mammo/1.0.0/. The INbreast dataset can be publicly accessible at https://www.kaggle.com/datasets/martholi/inbreast with a request to https://www.ncbi.nlm.nih.gov/pubmed/22078258

## Background

Breast cancer is the most prevalent cancer among women globally. For this cancer type, over 2.3 million newer cases and approximately 6,85,000 deaths globally were accounted in 2020 [1]. The golden standard for the initial screening and earlier detection is mammography imaging [2]. At the same time, its manual interpretation is time-consuming and easily vulnerable to inter-observer variability. Also, the mammographic interpretation for women with denser breast tissues [2] remains challenge. Computer-Aided Detection (CAD) techniques have emerged to assist radiologists in the accurate detection and diagnosis of breast cancer. These techniques aim to minimize large variations in interpretations. The development of these techniques aims to provide a promising and robust solution in interpreting the mammographic data [[3], [4]]. In terms of mammographic analysis, benign and malignant are the two basic severities of breast tumors. In this context, benign refers to non-cancerous growths. These are characterized by slower growth, a lack of invasion into surrounding tissues, and an inability to spread to other body parts. These benign tissues often possess smooth and regular, slightly deterministic shapes [5]. In contrast to benign cases, the malignant type characterizes cancerous tumors that usually have rapid growth with the ability of invading other tissues [5].

In the earlier research on CAD works, machine learning (ML) algorithms are equipped, such as Support Vector Machines (SVM) and Random Forest (RF). These traditional algorithms are applied to handcrafted features, but they lack robustness [6]. Several works [[7], [8], [9]] have been published with the adoption of handcrafted features using ML algorithms. In recent years, the advent of deep learning (DL) has revolutionized medical imaging and its analysis [6]. In particular, Convolutional Neural Networks (CNNs) enable end-to-end feature extraction from raw inputs. A detailed comparison between traditional ML and DL approaches in the context of mammography is presented in Table 1. For example, Wu et al [10] demonstrated AUC ∼0.895 using patch-level and image-level CNNs performing on par with experienced radiologists. Irrespective of CNNs’ efficiency, the standard CNN models may underperform in modeling long-range spatial dependencies [11]. Additionally, the CNN models have difficulty in interpreting denser tissue regions [11]. This remains a common challenge in mammographic image analysis and classification. ResNet, introduced by He et al. [12], improves representational depth and gradient flow through residual connections. Consequently, it is often a better choice for analyzing mammographic images. While performing the analysis of mammogram images, single-view images were widely used popularly. Based on this single-view consideration of mammogram image analysis, several research studies have evolved [[13], [14], [15]]. However, with the advent of multi-view consideration, the performance of breast cancer detection using mammograms has become better. In this concept, we are simply providing more details about the input mammograms to the classifiers as compared to the single-view analysis. Furthermore, multi-view architectures align better with clinical practice. For example, works [[16], [17], [18]] demonstrate that multi-view deep CNN-based classification outperforms single-view and feature-based classifiers. This improvement is achieved by incorporating both craniocaudal (CC) and mediolateral oblique (MLO) viewpoints.

**Table 1:** Comparison of traditional machine learning and deep learning approaches in mammography [[6], [7], [8], [9], [10]].

Feature	Traditional Machine Learning	Deep Learning Approach
Feature Extraction	Relies on manual feature engineering (e.g., texture, shape), which requires domain expertise.	Learns hierarchical features directly from raw images (End-to-End learning).
Robustness	Often struggles with variations in breast density and image quality.	More robust to noise and variations; capable of modeling complex, non-linear patterns.
Contextual Understanding	Typically analyzes local features in isolation.	Captures both fine-grained details and long-range spatial dependencies.
Performance	Larger datasets often result in a plateau effect and a higher incidence of false positives.	Efficiently handles large datasets, typically resulting in improved sensitivity and specificity.
Suitability for Mammography	Effective for simple, clear masses but struggles with dense tissue and architectural distortion.	Highly effective for detecting subtle lesions, microcalcifications, and abnormalities in dense tissue.

Additionally, attention mechanisms [19] demonstrated promising results in ultrasound and mammogram image segmentation tasks. For example, multi-attention frameworks [19] provided improved detection in mammograms and added interpretability in breast lesion classification. On the other hand, these architectures often relied on pre-trained models such as VGG16. This leads to a shortfall in the development of focused end-to-end designs specifically targeting mammography. Vision transformers have been applied to mammography at a larger scale. Chen et al. [19] employed multi-head self-attention on all four mammograms together. This shows that a transformer-based model that jointly processes the CC/MLO of both breasts can outperform state-of-the-art multi-view CNNs. These works demonstrate the significance of attention mechanisms (whether self-, spatial-, or channel-based). Specifically, they highlight the mechanism's role in focusing on critical image regions and modeling inter-view relationships in mammogram analysis.

**Fig. 1:**
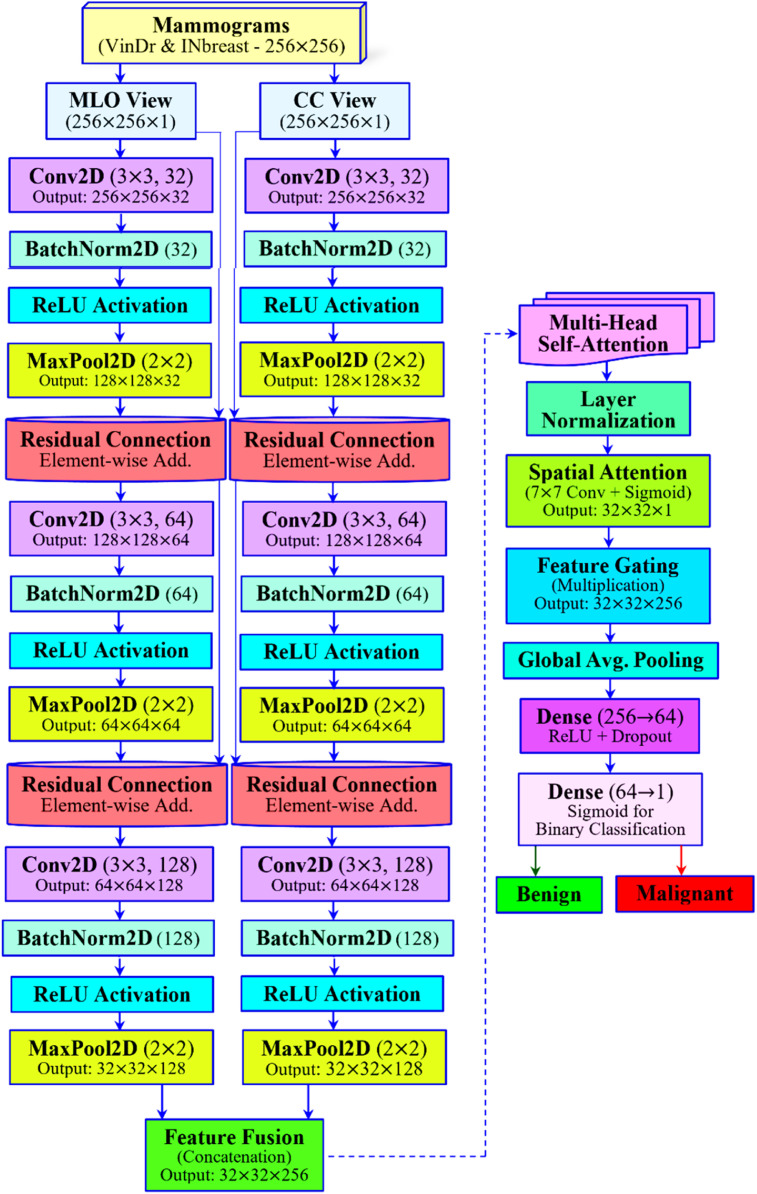
Proposed MammoFusion-Net architecture.

Besides the aforementioned attention mechanisms, explicit fusion strategies have been utilized to combine multi-view features. The recently developed PMVnet model proposed by Seo et al. [20] fuses paired views by making use of a U-Net-style architecture with cross-view modules. By leveraging this, the work computes inter-image feature similarity. Then, it incorporates squeeze-and-excitation (channel attention) to re-weight features from CC/MLO pairs. This results in much higher lesion segmentation/detection recall than single-view baselines. In a similar way, a gated attention fusion module in a multi-scale transformer model is introduced by Ahmed et al. [21]. This fusion module learns view-specific gating weights to dynamically integrate features from distinct mammogram images. Consequently, this process enhances the combined representation. In their evaluation, this fusion strategy outperformed simpler fusion schemes and attained a higher AUC (≈0.98) on a private BI-RADS dataset. In summary, recent works often incorporate learnable fusion layers. These layers utilize attention, gating, or similarity modules to adaptively merge CC and MLO features. And this becomes critical for effective cross-view breast cancer classification. Fig. 1 illustrates the proposed MammoFusion-Net architecture for the employed problem. An alternative to the existing architectures, the highlights of our proposed MammoFusion-Net architecture, as illustrated in Fig. 1, are as follows:

(i) The framework integrates residual CNN blocks, a multi-view dual-branch strategy, and multi-head self-attention. This combination facilitates deep feature learning and long-range dependency modeling.
(ii) For highlighting clinically relevant image regions, the framework incorporates spatial attention, and it further improves interpretability.
(iii) A Gated Cross-View Fusion mechanism is utilized to adaptively balance the contributions of each view. This approach provides a more effective fusion strategy compared to conventional concatenation.
(iv) Finally, the work demonstrates Grad-CAM-based explainability for aligning model focus with medical findings.

## Method details

The methods section describes the dataset selection and provides an elaborative examination of the proposed framework.

### Datasets used

**Fig. 2:**
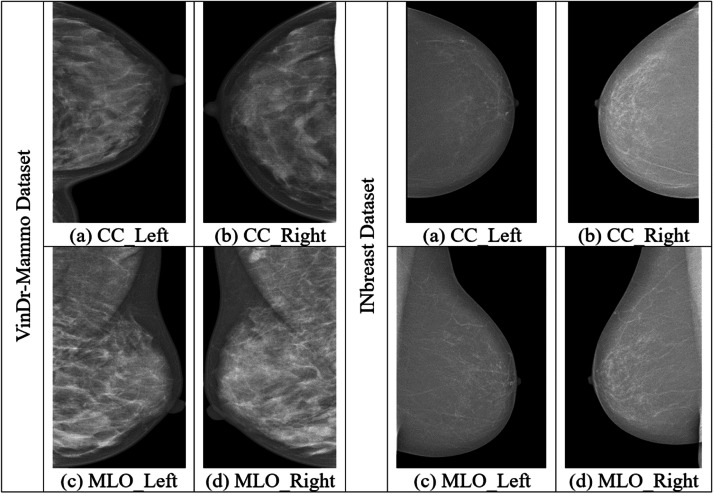
Sample mammograms of VinDr-Mammo and INbreast datasets.

As given in Fig. 1, the research employs two datasets, namely VinDr-Mammo [22] and INbreast [23] datasets. The reason behind selecting these datasets for evaluation is because of their high-quality mammogram images, i.e., Full-Field Digital Mammograms (FFDM). Here, the VinDr-Mammo is a larger-scale dataset containing higher-quality FFDM images curated for both screening and diagnosis of breast cancer. This dataset provides detailed radiologist-verified annotations, which include lesion locations and BI-RADS assessments [22]. This makes it a better choice for training and evaluating any AI-based diagnostic models. Apart from these, this dataset has the advantages of its clinical relevance, modern imaging format, and diverse population data. This made it suitable for enhancing the robustness and generalizability of ML and DL applications. In addition, the dataset consists of MLO and CC views for each breast. This enables the paper to perform a comprehensive multi-view analysis [22]. The work employs a subset of 2410 mammograms. Here, 1876 are benign, and 534 are malignant cases. The next one, although smaller in scale, is the INbreast dataset. This second dataset is widely used for the evaluation of DL architectures due to its higher-resolution FFDM images [23]. The work utilized a total of 150 mammograms from this database, where 76 benign and 74 malignant images were chosen. The INbreast dataset is smaller than the VinDr dataset. However, due to its higher annotation accuracy and image quality, it is employed in this work for further validation. Both the employed datasets contain mammograms in MLO and CC views, which makes them suitable for validating the proposed framework. The sample illustration of different views of mammograms of two datasets is given in Fig. 2.

### Data pre-processing and augmentation

In both FFDM datasets, each image was categorized as MLO or CC using the metadata provided with the datasets. Based on laterality, the MLO and CC views were paired for each breast. For maintaining input consistency across the dataset, the unpaired views were excluded. As mentioned in [24], for the preprocessing of mammograms, the Otsu thresholding [25] technique is applied for the extraction of the breast area. This method of basic mammogram processing works on the computation of the optimal threshold. This depends on either maximizing inter-class variance or minimizing intraclass variance. This method of thresholding is employed due to its effectiveness, simplicity, stability, and automatic threshold selection. For processing and pectoral muscle removal of MLO view mammograms, the algorithm in [26] is performed. Moreover, CLAHE - Contrast Limited Adaptive Histogram Equalization [27] is applied for improving the mammogram’s local contrast. To facilitate the proposed framework to have training with a more diverse range of mammogram inputs, data augmentation is performed. For this, the mammogram inputs undergo flipping and rotation with six distinct angles (45°, 90°, 135°, 180°, 234°, and 270°). This improves the model's generalization capability, and thus, the problem of overfitting has been effectively minimized in the risk of class imbalance. In addition, all mammographic inputs are resized to 256×256 prior to model input. While original FFDM images have much higher resolutions, this downsampling is necessary for computational efficiency. It significantly reduces memory usage and ensures training stability. For image-level classification, critical patterns such as tissue density and structural asymmetries are largely preserved at this resolution. Moreover, standardized input sizes facilitate consistent training and fair comparisons with baseline models. Furthermore, based on laterality, CC and MLO views from the same breast were paired (e.g., Left-CC with Left-MLO) to form a single input sample for the network. Cross-laterality pairings are not considered in the analysis. Table 2 summarizes the number of mammograms employed for the employed problem.

**Table 2:** Augmenting mammograms (training set) of two databases.

Labels	VinDr-Mammo Dataset			INbreast Dataset		
	Actual Images	Training Set		Actual Images	Training Set	
		Before Augmenting	After Augmenting		Before Augmenting	After Augmenting
Benign	1876	1313	10,504	76	52	416
Malignant	534	374	2992	74	51	408

### Proposed architecture: MammoFusion-Net with gated cross-view fusion

Fig. 1 illustrates the overall architecture of the MammoFusion-Net model. As given, the architecture is designed carefully for the extraction of both local and global features while ensuring efficient gradient flow and clinical interpretability.

*(a) Multi-View Dual-Branch Input Strategy*

The proposed MammoFusion-Net architecture starts with a design for explicitly handling the dual-view nature of mammographic images. Unlike single-view or naïve fusion models, the proposed MammoFusion-Net employs a multi-view dual-branch input strategy. This enables the architecture to process MLO and CC views independently through separate, view-specific convolutional streams. In this way, each breast’s MLO and CC views of two employed datasets are treated as paired inputs for preserving the spatial and anatomical diversity captured in each projection. Herein, the MLO view captures the upper outer quadrant and pectoral muscle. In contrast, the CC view emphasizes the central and lower breast regions, as portrayed in Fig. 2. Thus, the research emphasizes that processing them in a combined input space would dilute these critical anatomical distinctions. Here, the MLO and CC view mammograms are represented as I_MLO and I_CC. These dual-view mammograms are processed through two residual convolutional branches independently and are mathematically represented as given in Eq. (1).

(1) $$F_{MLO}=f_{\theta_{MLO}}\left(I_{MLO}\right)\;\text{and}\;F_{CC}=f_{\theta_{CC}}\left(I_{CC}\right)$$

In Eq. (1), $f_{\theta_{MLO}}$ and $f_{\theta_{CC}}$ represent the residual convolutional mappings with independently learned weights. Thus, these weights enable each branch to specialize in the unique spatial structures of its respective view.

*(b) View-Specific Residual Convolutional Branches*

The MammoFusion-Net begins with the independent processing of the MLO and CC view mammograms through two parallel residual convolutional branches. The mammogram inputs for both MLO and CC streams have dimensions of 256×256. As shown in Fig. 1, three sequential residual convolutional blocks are used to process each view. And this is essential for maintaining feature integrity throughout the network's depth. In Fig. 1, each branch of the first convolutional layer has the characteristics of a 3×3 kernel with 32 filters. This results in an output feature map of size 256×256×32. The fine-grained spatial features present in the mammogram inputs are captured by this layer. For stabilizing the learning process after convolution, batch normalization is applied. This is done by normalizing the activation distributions. Next, a ReLU activation function is utilized for introducing non-linearity. This enables the proposed architecture to capture complex patterns within the data. Subsequently, a max pooling operation is performed with a 2×2 kernel. This helps to reduce the computational burden progressively while increasing the receptive field. As a result, the spatial dimension is reduced to 128×128×32. This residual connection is established by adding the input feature map to the block’s processed output. This is mathematically represented as given in Eq. (2).

(2) y=F(x)+x

In Eq. (2), x is the original input to the block. Here, the non-linear transformation comprises the convolution, batch normalization, ReLU, and pooling operations, which are denoted as F(x). This means of identity mapping allows gradients to flow directly through the network. Moreover, this helps in mitigating the vanishing gradient problem and thus enables the construction of deeper, more robust networks. At the same time, this residual connection also facilitates retaining the fine structural details that may otherwise be lost in the deeper layers. In a similar way, the second residual block increases feature depth using the following characteristics: a 3×3 convolutional layer (64 filters), expanding to 128×128×64, followed by batch normalization, ReLU activation, and max pooling with a final reduction of 64×64×64. Herein, another residual connection is formed for combining the input and the transformed features. This ensures that mid-level spatial patterns are preserved alongside the newly learned features. Similarly, the third block further expands the feature space using a 3×3 convolutional layer (128 filters) to reach 64×64×128. This is followed by batch normalization, ReLU activation, and max pooling. This results in a final reduction of 32×32×128. Similar to the previous blocks, this block integrates a residual connection to combine the input with the output. This step preserves the integrity of high-level semantic features.

Residual connections are employed throughout these blocks. They are essential for enabling the model to learn deeper representations without performance degradation. This learning strategy facilitates the learning of both direct mapping and identity mapping. Thus, ensures that the network can effectively model complex patterns while retaining view-specific anatomical structures of mammographic image inputs. Finally, each branch produces a richer, multi-scale feature representation of its respective view. This also encapsulates essential local and global diagnostic information in a compact 32×32×128 feature map.

*(c) Gated Cross-View Fusion Technique*

**Fig. 3:**
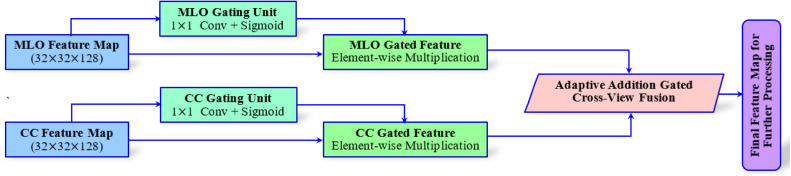
Gated cross-view fusion mechanism.

A Gated Cross-View Fusion mechanism is utilized to fuse the final feature map output obtained from the aforementioned MLO and CC branches (each of size 32×32×128). As compared to simple concatenation techniques [[27], [28]], the fusion technique adaptively weighs each view’s contribution based on its diagnostic relevance for each input. Fig. 3 portrays the workflow of the Gated Cross-View Fusion mechanism for the MammoFusion-Net framework. As shown, in this approach, a gating unit is used where each view-specific feature map is passed. This unit comprises a 1×1 convolutional layer followed by a sigmoid activation. This can be represented mathematically as shown in Eq. (3).

(3) $$g_{MLO}=\sigma \left(W_{g1}*F_{MLO}+b_{g1}\right),\;g_{CC}=\sigma \left(W_{g2}*F_{CC}+b_{g2}\right)$$

In Eq. (3), $W_{g1}$ and $W_{g2}$ represent the learnable weights, and σ is the sigmoid activation function. According to the input-specific diagnostic significance, the gating maps control each view’s contribution to the final fused representation. The gating maps are then applied to their respective feature maps through the operation of element-wise multiplication. This can be represented mathematically as shown in Eq. (4).

(4) $$F_{MLO}^{g}=g_{MLO}⊙F_{MLO},\;F_{cc}^{g}=g_{cc}⊙F_{CC}$$

Subsequently, the gated features are fused adaptively by summation as represented in Eq. (5).

(5) $$F_{fused}=F_{MLO}^{g}+F_{CC}^{g}$$

This fusion approach, as mentioned in Eqs. (3) to (5), enables the model to dynamically adjust the influence of each view on a per-sample basis. Thus, allowing the model to ensure that clinically significant features from either view are emphasized.

*(d) Multi-Head Self-Attention*

**Fig. 4:**
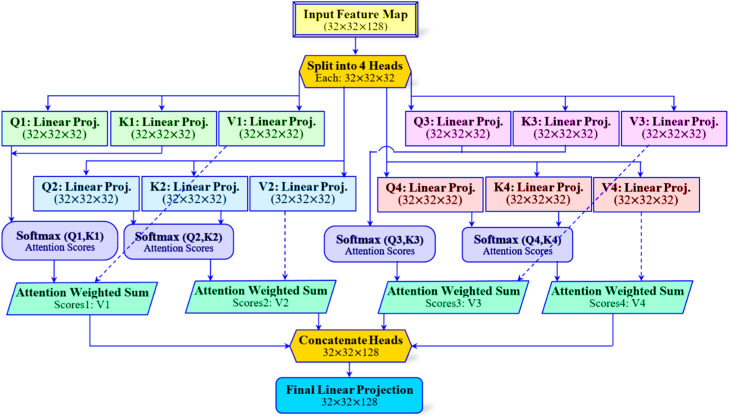
Multi-head self-attention (4-heads).

The feature maps after Gated Cross-View fusion with the size 32×32×128 are then passed through a multi-head self-attention block with four attention heads, as illustrated in Fig. 4. Herein, each head computes scaled dot-product attention and is mathematically represented as given in Eq. (6) [29].

(6) $$Attention\;\left(Q,K,V\right)=softmax\left(\frac{QK^{T}}{\sqrt{d_{k}}}\right)V$$

In Eq. (6), Q,K, and V represent the query, key, and value matrices, respectively, derived from the fused features, whereas $d_{k}$ denotes the key-dimension. As shown in Fig. 4, the outputs from the four heads are concatenated. Then they are projected back to the original channel dimension to form the attention-enhanced feature map. In this way, the multi-head self-attention mechanism enables the model to capture long-range spatial dependencies and context relationships. These relationships may span distinct, distant regions of the mammogram inputs. Subsequently, as given in Fig. 1, next to this attention, layer normalization is applied for ensuring stability during training and to normalize feature distributions.

*(e) Spatial Attention Module*

For further refining the feature map, the model applies a spatial attention module [30] as given in Fig. 1. This module is designed to emphasize spatially significant lesion regions while suppressing irrelevant background. For this, the module performs the computation of both global average pooling and global max pooling along the channel axis. Then, these pooled maps are concatenated as shown in Fig. 1. These maps are then passed through a 7×7 convolutional layer with a sigmoid activation function to produce the spatial attention map. This process is mathematically represented in Eq. (7).

(7) $$M_{s}=\sigma \left(f^{7\;\times \;7}\left(Concat\left[AvgPool\left(F_{fused}\right);MaxPool\left(F_{fused}\right)\right]\right)\right)$$

Subsequently, the spatial attention map is applied to the feature map using the operation of element-wise multiplication and is mathematically represented as given in Eq. (8).

(8) $$F^{\prime }=F_{fused}⊙M_{s}$$

The above step ensures that the MammoFusion-Net model focuses selectively on regions of the most informative lesion areas.

*(f) Classification Head*

The Global Average Pooling (GAP) [31] is then employed for processing the above-refined feature maps, and thereby a reduced 128-dimensional feature vector is obtained. This vector is then passed through a fully connected dense layer that reduces the dimension to 64 units. This is followed by ReLU activation and a dropout rate of 0.5 to mitigate overfitting. Finally, the output layer maps the 64-dimensional feature vector to a single output neuron with sigmoid activation, as shown in Fig. 1. This provides the malignancy probability mathematically represented in Eq. (9).

(9) $$\hat{y}=\sigma \left(W_{2}\;.\;ReLU\left(W_{1}\;.\;GAP\left(F^{\prime }\right)\right)\right)$$

In Eq. (9), $W_{1}$ and $W_{2}$ are weight matrices of the respective dense layers.

## Method validation

This section portrays the study’s experimentation, implementation, and outcomes of the proposed framework. The proposed MammoFusion-Net architecture is evaluated using two distinct mammogram datasets, namely VinDr-Mammo and INbreast FFDM images, after pre-processing. All experimentation in the research is conducted using stratified five-fold cross-validation. This ensures that the proposed framework has a balanced class distribution across the training and validation splits. This results in a robust and unbiased evaluation of the model’s classification ability for input mammograms. After CLAHE operation in the pre-processing phase, the partitioning of input mammograms is done stratified with a 70:30 ratio. Here, 70 % and 30 % are deliberated as training and testing inputs. Partitioning data prior to the augmentation process guarantees a precise evaluation. It ensures the proposed model's generalization capability is tested accurately. Therefore, the test data is retained unchanged to accurately reflect real-life scenarios. In such scenarios, the architecture must handle newer, unseen mammogram inputs. Additionally, statistical significance was assessed by comparing the performance of the proposed framework against baseline methods across repeated evaluations using hypothesis testing. Furthermore, it is to be noted that all evaluations are performed at the breast (case) level using paired CC–MLO inputs. The data splitting strategy ensured that both views associated with a given case were assigned to the same data partition, thereby preventing data leakage.

### Experimental outcomes and performance comparison

For model training, the work utilizes the Adam optimizer with $10^{-4}$ initial learning rate, 16 as batch size. Early stopping based on validation loss is adopted to mitigate overfitting. The learning rate was reduced by a factor of 0.1 when the validation loss plateaued for five consecutive epochs. For further regularizing the training, a dropout rate of 0.5 was incorporated in the fully connected layers. Each model was trained for a maximum of 100 epochs unless early stopping was triggered. As discussed in Section 2.1, the class imbalance problem of the employed datasets is addressed using the weighted binary cross-entropy loss function [32] as described in Eq. (10). This is specifically applied during the training phase to ensure that minority class samples contribute adequately to the optimization process.

(10) $$L_{weighted}=-\frac{1}{N}\sum_{i=1}^{N}w_{y_{i}}\left[y_{i}\log\left(\hat{y_{i}}\right)+\left(1-y_{i}\right)\text{log}\left(1-\hat{y_{i}}\right)\right]$$

In Eq. (10), $w_{y_{i}}$ denotes the inverse class frequency weight corresponding to each sample. This ensures that the minority class (malignant inputs), which are fewer in the dataset, makes an adequate contribution. Thus, they influence the optimization process effectively during model training. The proposed MammoFusion-Net architecture takes the image inputs of size 256×256. For comparative performance analysis, the evaluation employs the standard metrics for binary classification, namely Sensitivity (Sen), Specificity (Spc), Precision (Pre), Classification Error Rate (CER), Accuracy (Acc), AUC (Area Under the ROC Curve), and F1 Score [33]. Moreover, Cohen’s Kappa metric [33] is adopted for validating the obtained results.

Table 3 summarizes the ablation study experimentation for validating the significance of the individual module using the VinDr-Mammo dataset. Starting with the baseline model utilizing a dual-branch architecture with simple feature concatenation followed by classification, 74.274 % accuracy is achieved. This is due to its limited discriminative capacity when the model equally treats both views without any adaptive weighting. Next, when the Gated Cross-View Fusion is employed in place of simple concatenation, a substantial improvement of 81.051 % is obtained. This reveals the adaptive fusion’s significance in effectively prioritizing view-specific diagnostic information. For further improvement, the Multi-Head Self-Attention mechanism is incorporated, and so 85.754 % accuracy is obtained. This elevation is due to the capability of attention blocks. They capture long-range spatial dependencies and complex feature interactions, which are critical in mammogram analysis. As a final point, the Spatial Attention module is included in the model to make it focus on clinically significant regions. This results in 92.116 % accuracy for the proposed MammoFusion-Net model. This ensures that the contribution of Gated Fusion, Multi-Head Self-Attention, and Spatial Attention is significant for the proposed model’s classification capability. This demonstrates the importance of combining these blocks for robust classification performance.

**Table 3:** Ablation study for validating the impact of the individual module using VinDr-Mammo dataset.

Model Variant	Gated Fusion	Multi-Head Self-Attention	Spatial Attention	Accuracy (%)
Baseline: Dual-Branch + Concatenation	**✗**	**✗**	**✗**	74.274
+ Gated Cross-View Fusion	**✓**	**✗**	**✗**	81.051
+ Gated Fusion + Multi-Head Self-Attention	**✓**	**✓**	**✗**	85.754
Proposed MammoFusion-Net	**✓**	**✓**	**✓**	92.116

**Fig. 5:**
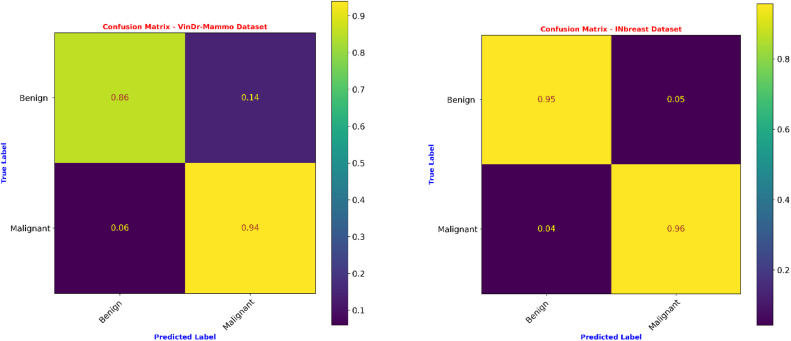
Confusion matrices of the proposed model for two datasets.

Fig. 5 gives the confusion matrices obtained for the Proposed MammoFusion-Net evaluated on VinDr-Mammo and INbreast datasets. Table 4 illustrates the comparative classification performance of MammoFusion-Net on the VinDr-Mammo dataset. It is compared against popular pre-trained models such as AlexNet, VGG16, VGG19, Inception-V3, and ResNet18. Herein, each architecture was tested under both mixed-view (single-branch) and multi-view (simple fusion) input configurations. The results reveal that multi-view processing across all architectures provided improved performance consistently as compared with mixed-view single-branch models. For example, an accuracy of 66.390 % with a sensitivity of 56.25 % and an AUC of 62.761 % is obtained for AlexNet in the mixed-view setting. When the same architecture was extended to a multi-view simple fusion strategy, performance improved. It reached 69.018 % accuracy and an AUC of 65.790 %. This demonstrates that even basic multi-view integration yields tangible outcomes. In a similar way, for VGG16 and VGG19 models, when moving from mixed-view to multi-view settings, the classification accuracy improved from 68.741 % to 70.124 % and from 71.923 % to 73.167 %, respectively.

**Table 4:** Comparative classification outcomes of the proposed MammoFusion-Net (VinDr-Mammo dataset).

Architectures	Sen (%)	Spe (%)	Acc (%)	Pre (%)	F1 Score (%)	CER (%)	AUC (%)
AlexNet (Mixed View) – Single Branch	56.25	69.27	66.390	34.22	42.55	33.61	62.761
AlexNet (Multi-View) – Simple Fusion	60.00	71.58	69.018	37.50	46.15	30.98	65.790
VGG16 (Mixed View) – Single Branch	59.38	71.40	68.741	37.11	45.67	31.26	65.389
VGG16 (Multi-View) – Simple Fusion	60.63	72.82	70.124	38.80	47.32	29.88	66.725
VGG19 (Mixed View) – Single Branch	61.88	74.78	71.923	41.08	49.38	28.08	68.326
VGG19 (Multi-View) – Simple Fusion	66.25	75.13	73.167	43.09	52.22	26.83	70.692
Inception-V3 (Mixed View) – Single Branch	70.00	78.86	76.902	48.48	57.29	23.10	74.432
Inception-V3 (Multi-View) – Simple Fusion	71.88	79.93	78.147	50.44	59.28	21.85	75.902
ResNet18 (Mixed View) – Single Branch	68.75	87.92	83.679	61.80	65.09	16.32	78.336
ResNet18 (Multi-View) – Simple Fusion	80.00	90.76	88.382	71.11	75.29	11.62	85.382
Proposed MammoFusion-Net (Multi-View with Gated Cross-View Fusion)	85.63	93.96	92.116	80.12	82.78	7.88	89.793

**Fig. 6:**
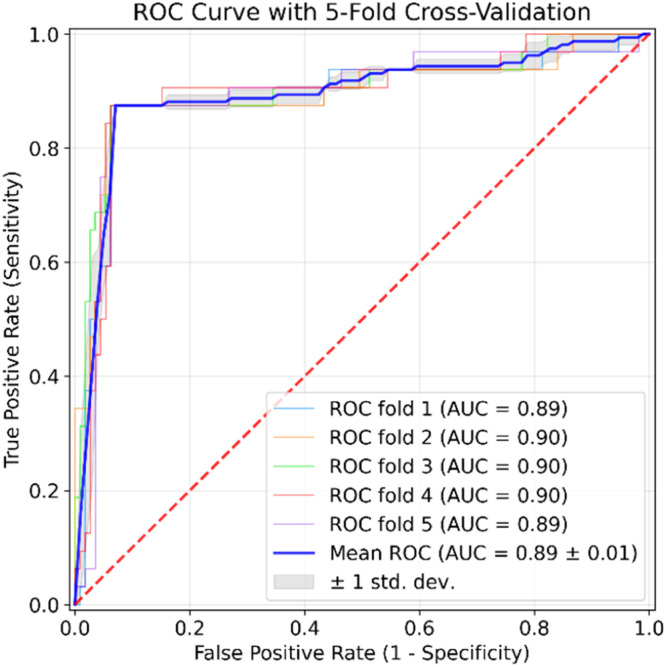
ROC plot of the proposed MammoFusion-Net (VinDr-Mammo) dataset using stratified 5-fold cross-validation.

These incremental outcomes validate the diagnostic value of leveraging both mammographic views (MLO and CC) for improved feature representation and deeper learning. In both view setups, Inception-V3 and ResNet18 models outperformed the AlexNet and VGG-based models consistently. In particular, ResNet18 (Multi-View) achieved the highest performance among the baseline models with 88.382 % accuracy, 80 % sensitivity, 11.62 % error rate, and an AUC of 84.53 %. This is due to the fact that deeper architectures with residual learning or inception modules exhibit improved classification performance in medical imaging tasks. In this way, the proposed MammoFusion-Net outperformed all comparative models substantially, as shown in Table 4. Specifically, MammoFusion-Net achieved an improved performance of 92.116 % accuracy. It also attained a sensitivity of 85.63 %, specificity of 93.96 %, precision of 80.12 %, and an F1 score of 82.78 %. Also, it is noted that a substantially lower classification error rate of 7.88 % and an AUC of 89.793 % are obtained. This indicates that the adaptive fusion strategy weighs view-specific features effectively and mitigates the limitations observed in simple concatenation-based fusion methods. To further evaluate the reliability of the observed performance improvements, statistical significance analysis is carried out. This is done by comparing the proposed framework with strong baseline methods. The resulting p-values indicate that the observed gains are statistically significant (p<0.05), suggesting that the improvements are unlikely to be due to random variation. As a final point, the results highlight that multi-view processing is essential for robust classification of mammogram severities. In addition to this, the proposed adaptive fusion strategy combined with attention mechanisms provides significant classification performance gains over traditional CNN architectures. Fig. 6 illustrates the ROC plot of the proposed MammoFusion-Net model. In order to evaluate the generalizability and robustness of the proposed MammoFusion-Net, the ROC curve generation involves stratified five-fold cross-validation. Herein, the ROC curve is first computed based on the test partition in each fold. In this way, the final ROC curve is obtained through averaging the true positive rate (TPR) and false positive rate (FPR) values across all five folds. In a similar manner, the mean AUC score is reported together with the standard deviation across folds. This way of computation ensures that the reported ROC curve of Fig. 6 is not biased by any single split of the dataset and thus reflects the model’s overall stability. The mean ROC curve is presented in Fig. 6, along with individual fold curves and a shaded confidence region (±1 standard deviation). The obtained area under the curve (AUC = 0.89±0.01) highlights the effectiveness of the fusion-based architecture across different data partitions. This validates the reliability of the framework, particularly in challenging diagnostic scenarios.

Similar to the VinDr-Mammo results, Table 5 illustrates the performance of MammoFusion-Net on the INbreast dataset. It compares the proposed model against standard pre-trained models like AlexNet, VGG16, VGG19, Inception-V3, and ResNet18. Herein, the Inception-V3 and ResNet18 models demonstrated stronger baseline performance as compared with AlexNet and VGG variants. In particular, 90.222 % accuracy, 89.73 % precision, and an AUC of 90.226 % are obtained for the ResNet18 (Multi-View – Simple Fusion) model. This supremacy of Inception-V3 and ResNet18 is due to their characteristics of effective residual learning and deeper feature representations in the employed mammogram classification problem. Similar to VinDr-Mammo dataset results, the highest sensitivity of 94.59 %, specificity of 96.49 %, accuracy of 95.556 %, precision of 96.33 %, and F1 score of 95.45 %. Also, the proposed model attains the lowest CER and highest AUC of 4.44 % and 95.543 %. This indicates that adaptive fusion and attention mechanisms significantly improved the discriminative power and robustness of MammoFusion-Net. Consequently, the model shows promise for breast cancer screening. Consistent with the findings on the VinDr-Mammo dataset, statistical significance analysis has been performed on the INbreast dataset. This confirms that the performance improvements achieved by the proposed framework are statistically significant (p<0.05). This supports the robustness of the proposed approach across different datasets.

**Table 5:** Comparative classification outcomes of the proposed MammoFusion-Net (INbreast dataset).

Architectures	Sen (%)	Spe (%)	Acc (%)	Pre (%)	F1 Score (%)	CER (%)	AUC (%)
AlexNet (Mixed View) – Single Branch	76.58	61.40	68.889	65.89	70.83	31.11	68.990
AlexNet (Multi-View) – Simple Fusion	78.83	63.60	71.111	67.83	72.92	28.89	71.213
VGG16 (Mixed View) – Single Branch	81.08	65.79	73.333	69.77	75.00	26.67	73.435
VGG16 (Multi-View) – Simple Fusion	83.33	72.37	77.778	74.60	78.72	22.22	77.851
VGG19 (Mixed View) – Single Branch	87.84	70.18	78.889	74.14	80.41	21.11	79.007
VGG19 (Multi-View) – Simple Fusion	89.19	74.56	81.778	77.34	82.85	18.22	81.875
Inception-V3 (Mixed View) – Single Branch	89.64	78.95	84.222	80.57	84.86	15.78	84.294
Inception-V3 (Multi-View) – Simple Fusion	92.34	83.33	87.778	84.36	88.17	12.22	87.838
ResNet18 (Mixed View) – Single Branch	90.09	76.75	83.333	79.05	84.21	16.67	83.422
ResNet18 (Multi-View) – Simple Fusion	90.54	89.91	90.222	89.73	90.13	9.78	90.226
Proposed MammoFusion-Net (Multi-View with Gated Cross-View Fusion)	94.59	96.49	95.556	96.33	95.45	4.44	95.543

**Fig. 7:**
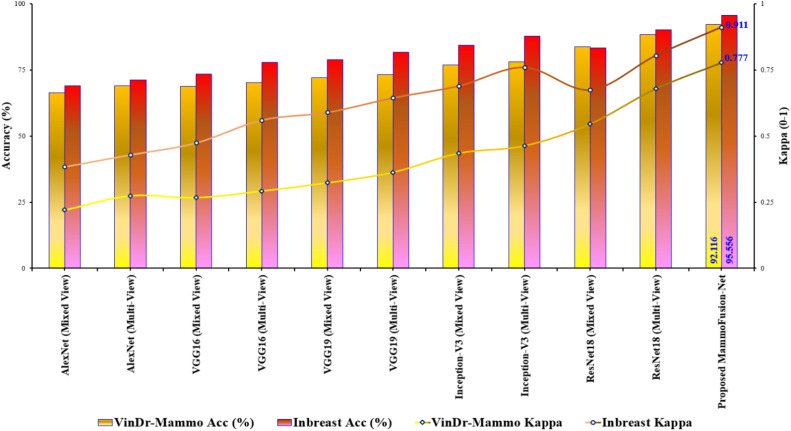
Comparative validation of the proposed model for VinDr-Mammo and INbreast datasets.

To validate these results for both datasets, Cohen’s kappa statistic metric is calculated. Fig. 7 illustrates the comparative validation of the proposed method for both datasets. Herein, the classification performance obtained for comparative models and the proposed method is validated. As a result of validation, the proposed method attains a validated higher kappa score of 0.777 (VinDr-Mammo) and 0.911 (INbreast). This further validates and confirms the supremacy of the proposed model for the employed classification problem. Additionally, it is well known that downsampling may reduce the visibility of very small lesions. However, the proposed framework is designed for image-level classification rather than precise localization. Furthermore, the observed performance indicates that the reduced resolution remains sufficient for effective classification.

### Grad-CAM-based interpretation

**Fig. 8:**
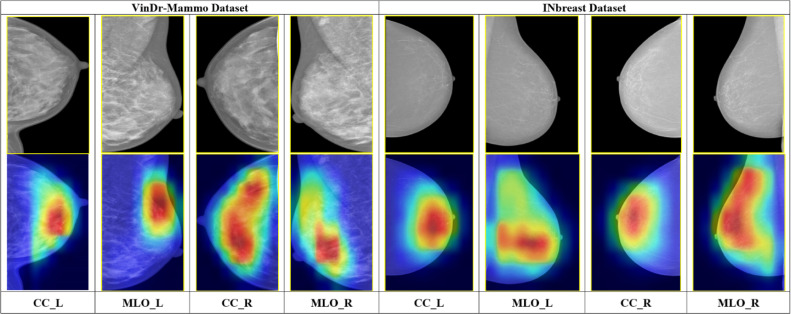
Sample Mammogram Examination (GradCAM): The gradient-based saliency maps highlight the most impactful pixels (depicted in red) that drive the proposed model's mammogram assessment.

**Fig. 9:**
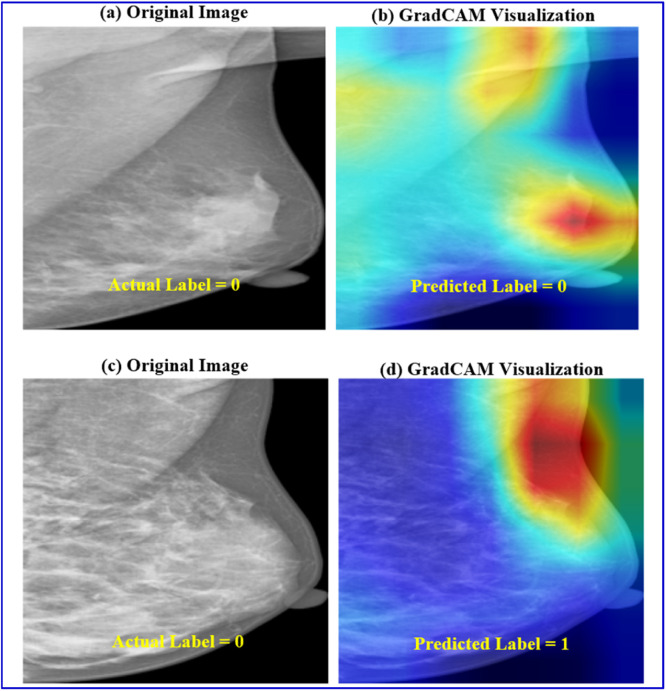
Sample Grad-CAM visualizations illustrating successful and failure cases. (a–b) Correct classification example where model attention aligns with visually prominent tissue regions associated with the annotated lesion. (c–d) Failure case where Grad-CAM highlights adjacent or diffuse regions, indicating partial misalignment with the annotated lesion.

The representative mammogram examinations using Grad-CAM [34] visualization are illustrated in Fig. 8. From both the VinDr-Mammo and INbreast datasets, the gradient-weighted class activation maps (Grad-CAM) for selected samples are portrayed. As shown in the Figure illustrating Grad-CAM visualizations, red-colored regions indicate areas of higher network activation. These regions contribute more strongly to the model’s classification decision. From a clinical perspective, the highlighted areas often correspond to localized tissue patterns. The examples for these patterns are dense structures or irregular intensity variations. Such features are commonly examined by radiologists during mammographic assessment. The model tends to focus on these visually prominent regions while suppressing homogeneous background tissue. This reveals that the network learns to emphasize anatomically relevant areas rather than irrelevant background information. Subsequently, this qualitative alignment with radiological reasoning enhances model interpretability without implying explicit lesion localization or diagnostic confirmation. Additionally, Fig. 9 presents sample Grad-CAM visualizations for both successful and failure cases. In the successful case, the highlighted regions generally correspond to visually prominent tissue areas. These regions typically overlap with or lie close to the annotated lesions. The model effectively focuses on denser or irregular structures. This can act as a key feature in mammographic assessment. In contrast, failure cases exhibit more diffuse attention. In these instances, the focus often shifts toward adjacent regions that do not fully align with the annotated lesions. This behavior reflects the image-level nature of the proposed framework. It also highlights the known limitations of Grad-CAM, which provides coarse localization rather than precise delineation. On the other hand, these visualizations offer qualitative insight into the decision-making process of the proposed model.

### Comparison with existing research studies

Table 6 illustrates a comparative analysis of the proposed MammoFusion-Net model against recent research studies on both the datasets (INBreast and VinDr-Mammo). In contrast to the listed works, the proposed MammoFusion-Net demonstrates consistent improvements over the existing frameworks for both datasets. These results reveal that the proposed model provides robust classification performance. Furthermore, it demonstrates robust generalization across varying dataset scales and characteristics.

**Table 6:** Comparison of the proposed model with existing research studies.

Reference	Framework	Accuracy (%)
Soulami et al. [35]	Apriori dynamics with Support Vector Machine (INBreast)	75.8
Dhungel et al. [36]	CNN with Pretraining Model (INBreast)	91
Shu et al. [37]	Neural Model with Region-Based Pooling (INBreast)	92.2
Zhang et al. [38]	Multi-Feature Fusion Model (INBreast)	85.37
Li et al. [39]	Convolutional Feature Descriptor Model (INBreast)	92.4
Liu et al. [40]	Deep Multi-scale Model with Regional Scoring (INBreast)	93.2
Hernández-Vázquez et al. [41]	Convolutional Neural Model (VinDr-Mammo)	73.0
Wen et al. [42]	Contrastive Joint Learning Network (VinDr-Mammo)	87.34
Proposed Model	MammoFusion-Net: INbreast Dataset MammoFusion-Net: Vin-Dr Mammo Dataset	95.556 92.116

## Limitations

• Although MammoFusion-Net achieved an improved classification performance and interpretability across two benchmark FFDM datasets, it has some limitations. First, the framework relies on paired CC-MLO views, which may not always be available in real-world screening. This might be due to missing or corrupted mammogram images. The proposed dual-branch strategy preserves view-specific features effectively. However, it does not explicitly model anatomical correspondences, which could further enhance fusion performance.
• Second, downsampling of input mammograms may reduce the visibility of very small lesions. This makes our future work to explore multi-scale or higher-resolution processing strategies. This will be done to further investigate the impact of image resolution on small lesion characterization and localization.
• As a final point, the evaluation was restricted to two datasets. Both are widely recognized benchmark datasets. However, broader validation on larger, multi-institutional datasets is necessary to confirm the model's generalizability. Additionally, cross-dataset evaluation was not explored in this study. Conducting such experiments is challenging due to differences in acquisition protocols and labeling strategies. Moreover, variations in data distributions between datasets may introduce domain shift. Future studies will specifically explore domain adaptation or harmonization techniques to address these challenges.

## Ethics statements

In this Manuscript no, human participants or animals their data or biological material, are not involved.

## CRediT author statement

***Sannasi Chakravarthy Surulimani Ramaraj*** *: Conceptualization, Methodology, Software Development, Data Curation, Formal Analysis, Writing – Original Draft.*

***Harikumar Rajaguru*** *: Investigation, Model Validation, Visualization, Writing – Review & Editing.*

***Rajesh Kumar Dhanaraj*** *: Supervision, Project Administration, Resources, Writing – Review & Editing.*

***Anto Lourdu Xavier Raj Arockia Selvarathinam*** *: Data Acquisition, Preprocessing, Software Implementation, Validation.*

***Dragan Pamucar*** *: Critical Review, Formal Analysis, Technical Guidance, Writing – Review & Editing.*

## Supplementary material *and/or* additional information [optional]

*NA.*

## Declaration of interests

The authors declare that they have no known competing financial interests or personal relationships that could have appeared to influence the work reported in this paper.

## Data Availability

Data will be made available on request.
